# Selenoprotein-Transgenic *Chlamydomonas reinhardtii*

**DOI:** 10.3390/nu5030624

**Published:** 2013-02-26

**Authors:** Qintang Hou, Shi Qiu, Qiong Liu, Jing Tian, Zhangli Hu, Jiazuan Ni

**Affiliations:** 1 Shenzhen Key Laboratory of Marine Biotechnology and Ecology, Department of Marine Biology, Shenzhen University, Shenzhen 518060, China; E-Mails: hqtdream2007@sina.com (Q.H.); jing.tianjingtj@gmail.com (J.T.); huzl@szu.edu.cn (Z.H.); 2 Shenzhen Key Laboratory of Microbial Genetic Engineering, College of Life Sciences, Shenzhen University, Shenzhen 518060, China; E-Mail: qiuqiushi@hotmail.com

**Keywords:** 15-KDa selenoprotein (Sep15), alga, *Chlamydomonas reinhardtii* (*C. reinhardtii*), selenium (Se), selenocysteine insertion sequence (SECIS)

## Abstract

Selenium (Se) deficiency is associated with the occurrence of many diseases. However, excessive Se supplementation, especially with inorganic Se, can result in toxicity. Selenoproteins are the major forms of Se *in vivo* to exert its biological function. Expression of those selenoproteins, especially with the application of a newly developed system, is thus very important for studying the mechanism of Se in nutrition. The use of *Chlamydomonas reinhardtii* (*C. reinhardtii*) as a biological vector to express an heterogeneous protein is still at the initial stages of development. In order to investigate the possibility of using this system to express selenoproteins, human 15-KDa selenoprotein (Sep15), a small but widely distributed selenoprotein in mammals, was chosen for the expression platform test. Apart from the wild-type human Sep15 gene fragment, two Sep15 recombinants were constructed containing Sep15 open reading frame (ORF) and the selenocysteine insertion sequence (SECIS) element from either human Sep15 or *C. reinhardtii* selenoprotein W1, a highly expressed selenoprotein in this alga. Those Sep15-containing plasmids were transformed into *C. reinhardtii* CC-849 cells. Results showed that Sep15 fragments were successfully inserted into the nuclear genome and expressed Sep15 protein in the cells. The transgenic and wild-type algae demonstrated similar growth curves in low Se culture medium. To our knowledge, this is the first report on expressing human selenoprotein in green alga.

## 1. Introduction

Selenium (Se) deficiency is associated with the occurrence of many diseases, such as cancer, cardiovascular disease, and Alzheimer’s disease [1,2,3]. Se exerts its biological functions mainly through selenoproteins [1,4]. The 15 KDa selenoprotein (Sep15) is one of the key selenoproteins modulating *in vivo* redox balance and participating in the formation of disulfide bonds during protein folding [5]. In the selenoprotein family, Sep15 is relatively small and widely distributed in most mammalian tissues [1], but its structure and function remain unclear. Thus, this protein was chosen for the test of selenoprotein expression in a new system—*C. reinhardtii*.

Resembling other selenoproteins, Sep15 has a special gene structure, wherein the active selenocysteine (Sec) is encoded by a traditional stop codon UGA in the open reading frame (ORF). In order to decode the in-frame UGA to a Sec residue, a special stem-loop structure, designated as Sec insertion sequence (SECIS) element, must be present in the 3′-untranslated region (UTR) of a eukaryotic selenoprotein gene to guide the Sec translation and incorporation into the protein [6,7].

It is known that yeast and land plants do not express selenoproteins [8]. Surprisingly, a variety of selenoproteins were found in algae such as *Ostreococcus tauri*, *Ostreococcus lucimarinus*, and *C. reinhardtii* [8,9,10]. *C. reinhardtii* was reported to contain 10 selenoproteins [11], including two Selenoprotein W homologs (SelW1, SelW2), two phospholipid hydroperoxide glutathione peroxidase homologs (PHGPx1, PHGPx2), two selenoprotein M homologs (SelM1, SelM2), selenoprotein T (SelT1), selenoprotein K (SelK1), thioredoxin reductase (TR1), and methionine sulfoxide reductase A (MsrA1). Among those selenoproteins, SelW1 and PHGPx1 are two highly expressed proteins in the *C. reinhardtii*. Those *C. reinhardtii* selenoprotein genes share common origins with their mammalian homologs [8]. Additionally, *C. reinhardtii* cells contain a Sec tRNA that specifically recognizes the UGA codon. Thus, it is reasonable to detect the possibility for *C. reinhardtii* to use the endogenous selenoprotein expressing system, including Sec tRNA, SECIS element and some trans-acting factors, to express heterogeneous selenoproteins.

Currently, the use of expressing exogenous protein in *C. reinhardtii* is only at the initial research stage. This expression system has the advantages of non-toxic, low cost and short growth cycle compared with other systems. Several proteins have been already expressed successfully in *C. reinhardtii* through either nuclear transformation or chloroplast transformation [12,13], which provide references for our present study. Hereby three forms of human Sep15 gene fragments were constructed and expressed in *C. reinhardtii*. To our knowledge, this is the first report about expressing a human selenoprotein in green alga.

## 2. Experimental Section

### 2.1. Materials

A cell-wall-deficient *C. reinhardtii* strain CC-849 was obtained from the *Chlamydomonas* Genetic Center of Duke University (Durham, NC, USA). The expression vector pH124 containing the Amp^+^ and zeocin resistance was self-stored in the Institute of Marine Biotechnology, Shenzhen University. The *E. coli* strain DH5α was preserved in our laboratory. pMD18-T vector and ExTaq enzyme were purchased from Takara (Dalian, China). Plasmid pMD18-T-Sep15 containing a human Sep15 gene was constructed previously in our laboratory. Rabbit polyclonal antibody against the *N*-terminal peptide of Sep15 was synthesized by Abmart (Shanghai, China). Pierce ECL detection kit for Western blot analysis was purchased from Thermo Fisher (Rockford, USA). 

Restriction enzymes, T4 ligase, DNA polymerase, plasmid purification kit, DNA purification kit, DNA gel extraction kit, RT-PCR reaction kit were purchased from Takara (Dalian, China). Genome extraction kit was purchased from Sbsgene (Shanghai, China). PCR primer syntheses and DNA sequencing were performed by Sangon Biotech (Shanghai, China). TRIzol reagent was from Invitrogen (Shanghai, China). All chemical reagents, except otherwise noted, were obtained from HS-Science (Shenzhen, China). Chemicals like hydrochloric acid, nitric acid, perchloric acid, potassium hydroxide, and potassium borohydride were superior grade of pure (GR), while others including sodium selenite were of analytical pure grade (AR).

### 2.2. Primer Design

The sequences of human Sep15 and *C. reinhardtii SelW1* genes were extracted from the NCBI database and analyzed for their SECIS elements with the online program SECISearch. Primers listed in Table 1 were designed by Primer 5.0 according to the published sequences. Two forward primers F_1_, F_2_ and reverse primers R_1_, R_2_ were designed to amplify the wild-type full-length gene (containing both ORF and SECIS), the ORF, and the SECIS element of Sep15, respectively. The primers F_w_ and R_w_ were designed to amplify the SECIS of *C. reinhardtii* SelW1, and primers F_ble_ and R_ble_ were used for the amplification of *ble* gene. The introduction of *Cla1* site and protection bases in both ORF and SECIS serves as an overlap sequence for constructing the human Sep15 recombinant.

**Table 1:** Primers designed in the paper ^a^.

name	primer sequence
F_1_	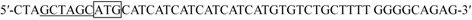
R_1_	5′-CCATCGATG GAAAAGGATAGGACAAAATTTAAGCA-3′
F_2_	5′-CCATCGATG GTACATAAAAACTTTGTAGCTTCATT-3′
R_2_	5′-ACGCGTCGAC ATCACTTTTAAATGGACTTTTCTGT-3′
F_W_	5′-CCATCGATG GACTGAGCACTGCCGCCCTGT-3′
R_W_	5′-ACGCGTCGAC GGCACAGCCTCATGACCTCCTA-3′
F_ble_	5′-GGCCAAGCTGACCAGCGCCGTTC-3′
R_ble_	5′-CTCCCGCCCCCACGGCTGCTC-3′

^a^ protection bases are in bold type. Restriction enzyme cutting sites are underlined including *Nhe* I (GCTAGC), *Cla* I (ATCGAT) and *Sal* I (GTCGAC). Start codon ATG is boxed off.

### 2.3. Plasmid Construction

*C. reinhardtii* CC-849 cells were cultured in tris-acetate-phosphate (TAP) medium to the late exponential growth phase. Approximately 5 × 10^7^ cells were harvested and washed twice with DEPC-treated H_2_O. Total RNA was extracted by Trizol reagent. The first-strand cDNA was reversed-transcripted using oligo-dT primers and ReverTra Ace reverse transcriptase (Toyobo, Japan) at 42 °C for 20 min, 99 °C for 5 min, and 4 °C for 5 min. The cDNA was used as a template to amplify the SECIS element of *C. reinhardtii* SelW1 with the primers F_w_ and R_w_ under the following PCR conditions: 30 cycles of denaturation at 94 °C for 30 s, annealing at 55 °C for 30 s and extension at 72 °C for 40 s.

Plasmid pMD18-T-Sep15 containing human Sep15 gene was used as a template for PCR amplification of the full-length Sep15 (primers F_1_, R_2_), its ORF (primers F_1_, R_1_), and its SECIS element (primers F_2_, R_2_). PCR was performed under the following reaction conditions: preheating at 94 °C for 5 min, 25 cycles of circulation at 94 °C for 30 s, 59 °C for 30 s, and 72 °C for 40 s, extension at 72 °C for 7 min.

The DNA fragments of Sep15 ORF and SECIS elements (from either *C. reinhardtii* SelW1 or human Sep15 genes) were used as templates and amplified by overlapping PCR to construct two recombinants of human Sep15 ORF plus *C. reinhardtii* SelW1 SECIS (primers F_1_, R_w_) and human Sep15 ORF plus SECIS (primers F_1_, R_2_). PCR conditions: preheating at 94 °C for 5 min; 5 cycles of circulation at 94 °C for 30 s, 42 °C 30 s, and 72 °C 40 s; addition of each pair of primers and running another 20 cycles of circulation at 94 °C for 30 s, 55 °C 30 s, and 72 °C 40 s; extension at 72 °C for 7 min. 

Three PCR products of wild-type and two recombinant Sep15 fragments were inserted into the pMD18-T vector respectively, and transformed into CaCl_2_ competent *E. coli* (DH5α strain) cells for ampicillin selection. The isolated plasmids were checked by PCR amplification and enzymatic digestion (with *Nhe* I and *Sal* I). Those Sep15-containing plasmids, including the human Sep15 ORF plus SECIS (pMD18-T-Sep15ORF-hSECIS), the human Sep15 ORF plus *C. reinhardtii* SelW1 SECIS (pMD18-T-Sep15ORF-chSECIS), and wild-type human Sep15 fragment (pMD18-T-wtSep15), were sequenced in Sangon Biotech (Shanghai, China).

Those sequence-confirmed Sep15-containing plasmids were then digested with *Sal* I restriction enzyme, purified, and the sticky ends were filled into blunt ones by two cycles of PCR using *Pfu* DNA polymerase without primers. After purification, the products were digested with *Nhe* I restriction enzyme to possess 5′-stick and 3′-blunt ends. The expression vector pH124 was then digested with the restriction enzymes *Nhe* I and *Pma*C I to get the same ends, so that those Sep15 fragments were inserted into the pH124 plasmids respectively. The constructed plasmids were transformed into *E. coli* cells, and PCR detection was performed to confirm the successful insertion. Three Sep15 expression plasmids were therefore constructed, including pH124-Sep15ORF-hSECIS, pH124-Sep15ORF-chSECIS, and pH124-wtSep15.

### 2.4. Nuclear Transformation of *C. reinhardtii*

Monoclonal CC-849 cells were inoculated into sterile TAP liquid medium and cultured to mid-logarithmic phase (approximately 1–2 × 10^6^ cells/mL) in the incubator under the following conditions: temperature, 25 °C; brightness/darkness ratio, 16/8 h; light intensity, 6000 l×. In order to achieve high transformation efficiency, three Sep15 expression plasmids were *Not* I-digested into linear plasmids before they were transformed into CC-849 cells by the glass-bead method [13]. Briefly, CC-849 cells in the mid-log phase were harvested by centrifugation at 5000 rpm for 5 min, suspended with fresh TAP, and adjusted to 2 × 10^8^ cells/mL. 300 μL cell suspension was transferred into EP tubes containing 0.5-mm-diameter sterilized glass beads. 2–5 μg linearized Sep15-containing pH124 plasmids were then added. A “no DNA” control was set up. The cell/glass bead/DNA suspension was shaken in vortex for 18 s at top speed, transferred into 20 mL TAP medium, and cultured for about 25 h by shaking at 100 rpm. The cells were centrifuged down, gently resuspended in TAP medium, mixed with 0.5% molten agar in TAP (below 42 °C, no antibiotic), and rapidly poured onto 2% TAP-agar plates containing 10 μg/mL zeocin. The plates were inverted and left under the light (6000 l×) at 25 °C. Green colonies were visible after 2–3 weeks.

### 2.5. Identification of Human Sep15 Fragments in *C. Reinhardtii*

The transgenic *C. reinhardtii* cells were harvested and genomic DNA was extracted for with the genomic extraction kit. PCR was performed using the genome as a template to check the presence of *ble* gene fragment (about 464 bp) with the primers F_ble_ and R_ble_, or to check the presence of Sep15 fragments: primers F_1_ and R_2_ for wtSep15 (about 1164 bp) and Sep15ORF-hSECIS (about 681 bp), primers F_1_ and R_w_ for Sep15ORF-chSECIS (about 690 bp). Reaction conditions: 95 °C for 5 min; 95 °C for 30 s, 55 °C for 30 s, 72 °C for 40 s; 30 cycles; 72 °C extension for 7 min.

To analyze the mRNA expression level, the transgenic algae were cultured to mid-log phase, heat-shocked for 20 min at 42 °C to promote exogenous gene transcription, and then put back to the incubator to grow for another 1–2 h. Total RNA were extracted from the cells with TRIzol. RT-PCR was performed using the same pairs of primers described in the genomic DNA amplification for wtSep15, Sep15ORF-hSECIS, and Sep15ORF-chSECIS, respectively. RT conditions: 30 °C, 10 min; 42 °C, 30 min; 99 °C, 5 min; 4 °C, 5 min. PCR conditions: 94 °C 5 min; 94 °C 1 min, 55 °C 30 s, 72 °C 40 s, 30 cycles; 72 °C 7 min. 

To analyze the expression of human Sep15 protein, the transgenic algae were grown in the medium supplemented with Na_2_SeO_3_ to a final concentration of 10 μmol/L. Both the transgenic and wild-type *C. reinhardtii* cells were collected by centrifugation. Cold acetone was added to the cells and stored at −20 °C overnight to remove the pigment. The cells were then centrifuged and washed twice with cold acetone. After complete evaporation of acetone on ice, the algal pellets were lysed in cell lysis solution by ultrasonication. After centrifugation, the supernatants were separated by SDS-PAGE and analyzed for Sep15 expression by Western blot using a rabbit polyclonal antibody against Sep15 (1:50 dilution). The immune complexes were revealed by enhanced chemiluminescence with the Pierce ECL detection kit. 

### 2.6. Evaluation of Alga Growth

Alga growth was evaluated by its cell density and dry-weight. The wild-type and transgenic *C. reinhardtii* were inoculated separately into 250 mL TAP medium containing Na_2_SeO_3_ at a series of final concentrations of 0, 5, 10, 15, and 20 μmol/L, each in triplicates. Cell density was detected every 24 h by a spectrometer at 650 nm wave-length (A_650_). To measure alga dry-weight, an aliquot of 50 mL cell culture was collected after growing for 24 h, followed by a 20 mL-collection at 48, 72, and 96 h, respectively. Those aliquots of cell cultures were harvested at 5000 rpm for 5 min, transferred to pre-weighed EP tubes, freeze-dried to a constant weight, and weighed on an analytical balance. Data were expressed as the mean ± SD of triplicate samples.

### 2.7. Se Measurement

Se in the algae was analyzed using the method of hydroxide generation-atomic fluorescence spectrometry (HG-AFS) [14,15]. Briefly, 0.02 g dry-algae was accurately weighed, digested with mixed acid (HNO_3_/HClO_4_, v/v = 4:1), reduced by 50% HCl, filled with 5% HCl solution to the final volume, and measured by an atomic fluorescence spectrometer (AFS-920, Jitian Instrument Co., Beijing, China). Instrument running condition: carrier solution, 5% HCl; reducing agent, 2% NaBH_4_; lamp current, 80 mA; negative voltage, 270 V. Analytical pure sodium selenite was used to prepare a stock solution of 10 ng/mL Se. A series of standard solutions at 1, 2, 4, 6, 8 and 10 ng/mL were diluted automatically from the stock solution for calibration curve. Each sample was performed in independent triplicates. Statistical analysis was performed using statistical software SPSS 11.5. *P* < 0.05 was considered as significant difference. Data were expressed as the mean ± SD of triplicate samples.

## 3. Results

### 3.1. Construction of Three Types of Human Sep15 Expression Plasmids

**Figure 1:**
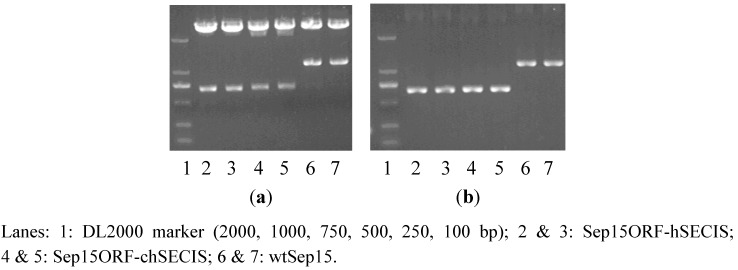
Detection of three types of Sep15 fragments byplasmid PCR analysis (**a**) andenzymatic digestion (**b**).

RT-PCR was performed to amplify the SECIS element of *C. reinhardtii* SelW1 (219 bp). Full-length human Sep15 gene (1164 bp), together with its ORF (482 bp) and SECIS element (209 bp), were amplified from the self-constructed plasmid pMD18-T-Sep15. During PCR-amplification, an overlapping DNA sequence, containing a *Cla* I restriction site and several protection bases, was introduced into the 3′-end of Sep15 ORF and the 5′-ends of two SECIS elements, which facilitated the operation of overlap PCR to get the recombinants of Sep15ORF-hSECIS (681 bp) and Sep15ORF-chSECIS (690 bp). The full-length Sep15 (wild-type Sep15, wtSep15) and two Sep15 recombinants were then inserted respectively into the pMD18-T vector and transformed into *E. coli* DH5a. Plasmids isolated from the *E. coli* cells were checked by PCR amplification and restriction enzyme digestion (Figure 1) for the proper molecular sizes of three types of Sep15 inserts. Those Sep15 inserts were further confirmed by DNA sequencing to be 100% correct (shown in the Supplementary Data). The wtSep15 fragment contains the start codon ATG, Sec-decoding TGA, stop codon TAA, and SECIS element in the 3′-UTR, which are boxed off in the Supplementary Data. The sites of restriction enzymes, including *Nhe* I (GCTAGC), *Cla* I (ATCGAT) and *Sal* I (GTCGAC), are underlined. 

The wtSep15 and Sep15ORF-hSECIS fragments have the same ORFs and SECIS elements of human Sep15 gene, except the distance between the stop codon TAA and SECIS element. For the fragments of wtSep15, Sep15ORF-hSECIS and Sep15ORF-chSECIS, those distances between the stop codon and SECIS element are 574, 94 and 107 nt, respectively. The distances in the two recombinants are similar, which were designed according to that of *C. reinhardtii* SelW1 gene, in order to use the algal selenoprotein translation system to express human Sep15. Sequence analysis with the SECISearch program showed that the secondary structures of SECIS elements from the mRNAs of human Sep15 and *C. reinhardtii* SelW1 have the same conserved pattern AUGA_AA_GA, a characteristic of eukaryotic selenoprotein SECIS element. Such similarity lays the foundation for the exchange of two SECIS elements for heterogeneous expression of human Sep15 in *C. reinhardtii*.

The pH124 expression vector contains the multiple-promoter Hsp70-RBCS2, which can be heat-activated and light-induced for the expression of exogenous genes in *C. reinhardtii*. However, the presence of several *Sal* I cutting sites limits the usage of this enzyme for the vector. Thus, the obtained Sep15 fragments were firstly digested with *Sal* I, and then the sticky ends were filled, followed by further digestion with *Nhe* I to obtain the fragments containing 5′-sticky and 3′-flat ends. The expression vector pH124 was also cut with *Nhe* I and *Pma*C I to generate the same ends. The Sep15 fragments were then ligated with the pH124 plasmids, and transformed into competent *E. coli* cells. Successful insertion of each Sep15ORF-hSECIS, wtSep15, and Sep15ORF-chSECIS into the pH124 vector was detected by plasmid PCR. 

### 3.2. Identification of Human Sep15 in *C. reinhardtii*

Plasmid-transformed algae were selected by the zeocin-containing plates due to the expression of *ble* gene constructed in the pH124 vector. CC-849 cells and the no-*ble-*plasmid transformed cells are very sensitive to zeocin that they could not grow on the zeocin-containing plate. Positively transformed cells, including pH124-wtSep15, pH124-Sep15ORF-hSECIS, pH124-Sep15ORF-chSECIS, and vacant pH124 transformed cells, expressed Ble protein to resist zeocin, leading to the growth of *C. reinhardtii* on zeocin-containing plates. 

**Figure 2:**
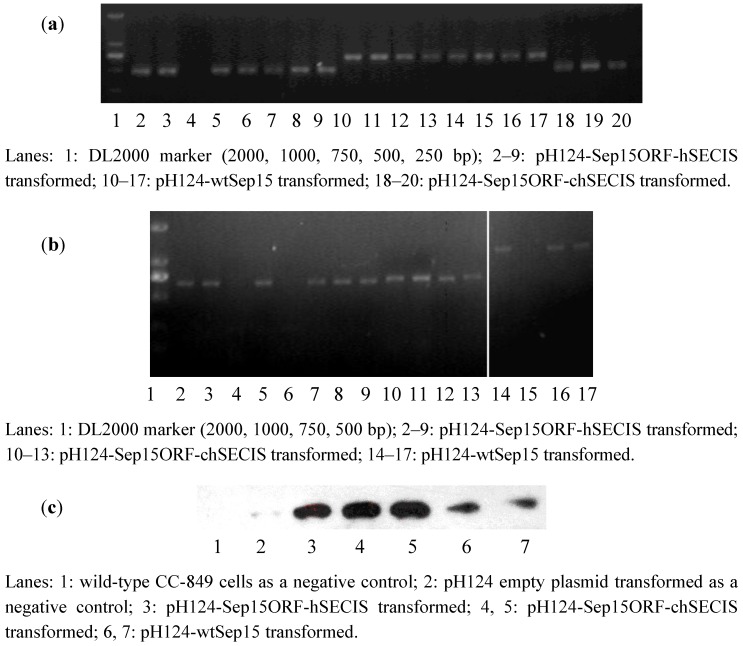
Detection of human Sep15 in the transformed *C. reinhardtii* CC-849 cells by genomic PCR (**a**), RT-PCR (**b**), and Western blot (**c**) analyses.

To investigate if human Sep15 fragments had been inserted into the genomes of CC-849 cells, genomic DNAs were extracted from the monoclonal algae grown on the zeocin-containing plates and medium. Genomic PCR was performed to identify positive transformants via the detection of *ble* gene and the fragments of wt-Sep15, Sep15ORF-hSECIS, and Sep15ORF-chSECIS. As shown in Figure 2a, lanes 2–9 contained the ORF fragments (482 bp) amplified from the pH124-Sep15ORF-hSECIS transformed algae. Seven out of eight clones in the corresponding plates had their Sep15 ORFs integrated into the genomes of CC-849 cells. Lanes 10–17 contained the Sep15ORF-chSECIS fragments (690 bp) amplified from the genomes of pH124-Sep15ORF-chSECIS transformed algae, and lanes 18–20 contained the Sep15 ORFs amplified from the pH124-wtSep15 transformed algae. All those clones had their Sep15 fragments positively integrated into the algal genomes.

RT-PCR was performed to investigate the expression of human Sep15 mRNA in *C. reinhardtii*, as shown in Figure 2b. Expression of human Sep15 mRNA was detected in six out of eight clones transformed with pH124-Sep15ORF-hSECIS plasmids, or four clones of the pH124-Sep15ORF-chSECIS transformed algae, or three out of four clones of the pH124-wtSep15 transformed algae.

Those positive clones detected by the genomic PCR and RT-PCR assays were selected for Sep15 protein expression. Sodium selenite was supplemented into the culture medium. Human Sep15 was induced to express in *C. reinhardtii* by heat-shock and light-induction due to the multiple promoters Hsp70-RBCS2. Algal pigment was removed before protein extraction and Western blot analysis. Wild-type *C. reinhardtii* and empty plasmid pH124 transformed *C. reinhardtii* were used as negative controls. As shown in Figure 2c, human Sep15 protein was detected to be expressed successfully in all types of transgenic *C. reinhardtii* cells, but not in two negative controls (Lanes 1 & 2). Those results showed that all positive transformants, including pH124-wtSep15, pH124-Sep15ORF-hSECIS, and pH124-Sep15ORF-chSECIS, could express human Sep15 in the *C. reinhardtii* CC-849 cells.

### 3.3. Cell Viability of Human Sep15-Transgenic *C. reinhardtii*

**Figure 3:**
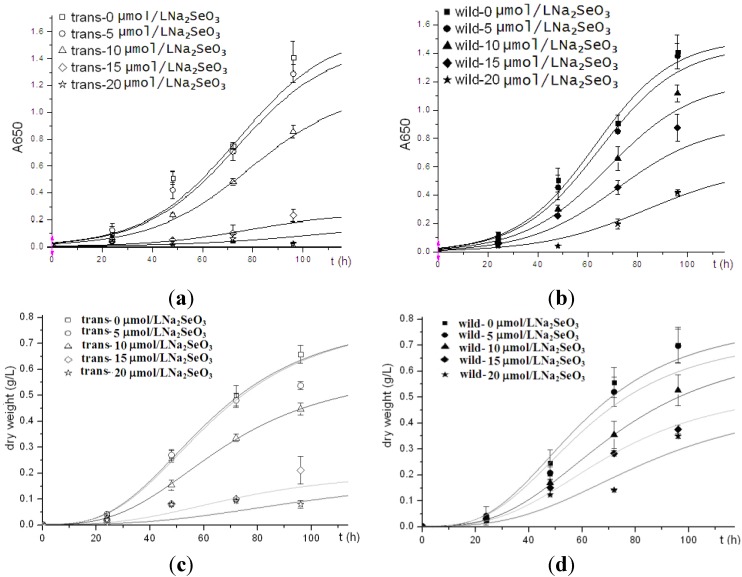
Growth curves of human Sep15 transgenic (**a**, **c**) and wild-type (**b**, **d**) *C. reinhardtii*.

**Figure 4:**
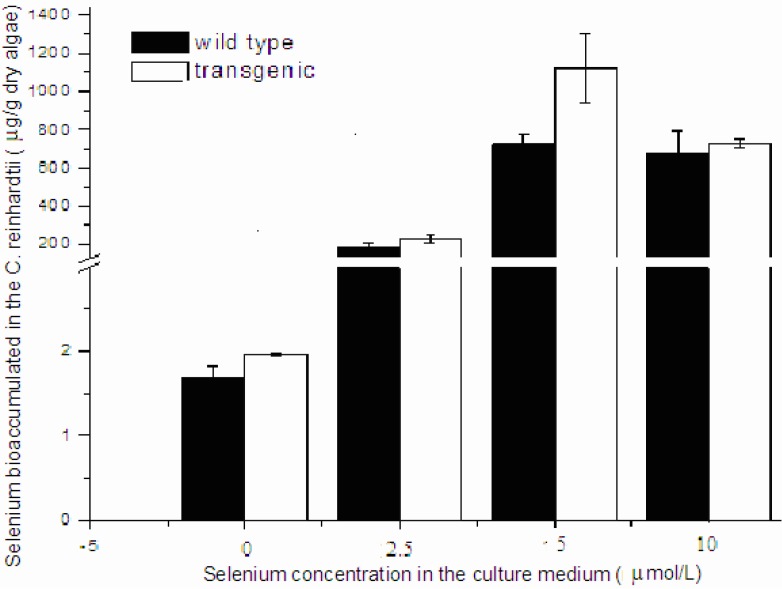
Se bioaccumulation in the transgenic and wild-type *C. reinhardtii*.

To compare the cell viability between human Sep15-transgenic and wild-type *C. reinhardtii*, the same amount of cells of both the transgenic and wild-type algae were inoculated into TAP medium supplemented with sodium selenite. Two types of algal growth curves were plotted according to their time-dependent changes in cell density (Figure 3a,b) and dry weight (Figure 3c,d). The transgenic and wild-type algae showed similar viability when they were cultured without selenite or under low concentrations of selenite (<10 μmol/L). These indicated that the insertion of human Sep15 gene into algal genomes and the expression of exogenous Sep15 proteins in the cells did not affect the viability of *C. reinhardtii*. The transgenic algae grew well and produced human selenoprotein Sep15 when the TAP medium was supplemented with limited amount of selenite. The Se levels of the algae initially increased with the Se concentration in the culture medium (<10 μmol/L), but reached a steady stage for the wild-type or started to decrease for the transgenic at higher concentrations (≥10 μmol/L) (Figure 4). When they were cultured in 5 µmol/L Se medium, the average level of Se was measured as 1100 μg/g dry alga for the transgenic (*n* = 3) and 733 μg/g dry alga for the wild-type (*n* = 3). Although the Se level of transgenic alga was slightly higher than that of wild-type, no significant difference was observed in the present study. Significant inhibition was found in the transgenic alga treated with 15 μmol/L selenite.

## 4. Discussion

For a eukaryotic selenoprotein mRNA, a traditional stop codon UGA is located inside its ORF and decoded as a Sec residue under the guidance of an essential SECIS element in the 3′-UTR. Since different SECIS elements from various species may have different efficiency, three types of Sep15 with different SECIS elements were designed and constructed in this paper. As for human Sep15 mRNA its in-frame UGA codon is located in the middle of ORF and two SECIS-like structures are in the 3′-UTR. The first SECIS-like structure does not function, only the second one guides the translation [16]. Therefore, a recombinant Sep15 fragment (Sep15ORF-hSECIS) was constructed to combine human Sep15 OFR directly with the second SECIS element, deleting the first SECIS-like structure in the wild-type. Results in this paper showed that both the wild-type and recombinant transformants could express human Sep15 in *C. reinhardtii*, which supported the opinion that the translation of UGA to Sec in Sep15 gene is only dependent on the second SECIS element. 

*C. reinhardtii* has been reported to contain ten selenoprotein genes in its genome, except for Sep15 [8]. As SelW1 is one of the two major selenoproteins highly expressed in *C. reinhardtii*, it is reasonable to deduce that the SelW1 SECIS element has high efficiency in guiding the UGA-to-Sec translation. Although each eukaryotic selenoprotein gene has its own specific SECIS element, replacement by other eukaryotic SECIS elements generally does not disturb the decoding of UGA to Sec [17]. In the present study, the SECIS element of *C. reinhardtii* SelW1 was combined with human Sep15 ORF to construct a recombinant of Sep15ORF-chSECIS, in order to test algal SECIS element for human Sep15 expression. The distance between the in-frame TGA and SECIS in the recombinant was designed to resemble that in the *C. reinhardtii* SelW1 gene, in order to reduce distance impact on Sep15 expression efficiency [18]. Results demonstrated that the SECIS elements of both *C. reinhardtii* SelW1 and human Sep15 were capable of guiding the expression of human Sep15 in *C. reinhardtii*. 

Land plants have lost their ability to synthesize selenoproteins during the evolution process [19]. However, sea plants like *C. reinhardtii* still keep this function to maintain life. The present study makes an effort to express a small selenoprotein in the algal, a new protein expression system. Improvement is in urgent need for getting high yield and expressing other selenoproteins with this platform. Apart from Sep15 expression, the transgenic alga has slightly higher Se level than the wild-type when they were exposed to 5 μmol/L sodium selenite. This level of Se in the transgenic algae is close to that of multiple-step Se-enriched algae (1301 μg/g dry algae) [20]. 

Maintaining Se at its physiological level is very important for human health. At least 40 μg/day Se supplement is essential for adults to maintain the expression and function of selenoproteins, and 300 μg/day for cancer treatment [21]. The *in vivo* function of Se is not only dependent on its level, but also on its form [22,23]. Organic Se generally has low toxicity and high bioavailability compared with inorganic Se. Agronomic biofortification and genetic improvement are potential solutions to increase organic Se in food. Animals, plants and microorganisms grown in Se-rich environment accumulate high level of organic Se [23,24]. However, those Se-enriched species can not highly express any specific selenoprotein. Meanwhile, Se binds non-specifically with abundant proteins in those enriched-species [25]. Thus, constructing transgenic species is a good way for improving Se bio-efficiency. *C. reinhardtii* is a non-toxic, edible alga containing many selenoproteins [9,10]. The Sep15 transgenic *C. reinhardtii* constructed in this paper improves the nutritional quality of this alga, making it a good and safe Se supplement for human health. It also provides a potential platform for high expression of recombinant selenoprotein in the green alga.

## 5. Conclusions

Three types of human Sep15 gene fragments, including Sep15ORF-hSECIS, Sep15ORF-chSECIS, and wtSep15, were successfully constructed and transformed into *C. reinhardtii*. All of them were detected to be integrated into the genome of CC-849 cells, and expressed human Sep15 in both mRNA and protein levels. The transgenic algae demonstrated similar growth curves to that of the wild-type when they were cultured in low Se media. Results in this paper indicate that the transgenic *C. reinhardtii* has potentiality to become a good Se-supplement and a host to express exogenous selenoproteins.

## Supplementary Files

Supplementary File 1 Supplementary Data (PDF, 11 KB)
