# Supplementary material for: Selenoprotein-Transgenic Chlamydomonas reinhardtii
Source: Nutrients. 2013 Feb 26;5(3):624–36. doi: 10.3390/nu5030624 (PMC3705309; doi:10.3390/nu5030624)
Supplement: Supplementary File 1 — Supplementary Data (PDF, 11 KB) [file nutrients-05-00624-s001.pdf]

Supplementary Data

Primary sequences of three types of Sep15 fragments:

(a) wtSep15

TAGCTAGCATGCATCATCATCATCATCATGTGTCTGCTTTTGGGGCAGAGTTTTCATCGGAGGCATGCAGAGAGTTAGGCT
TTTCTAGCAACTTGCTTTGCAGCTCTTGTGATCTTCTCGGACAGTTCAACCTGCTTCAGCTGGATCCTGATTGCAGAGGATG
CTGTCAGGAGGAAGCACAATTTGAAACCAAAAAGCTGTATGCAGGAGCTATTCTTGAAGTTTGTGGATGAAAATTGGGA
AGGTTCCCTCAAGTCCAAGCTTTTGTTAGGAGTGATAAACCCAAACTGTTCAGAGGACTGCAAATCAAGTATGTCCGTGG
TTCAGACCCTGTATTAAAGCTTTTGGACGACAATGGGAACATTGCTGAAGAACTGAGCATTCTCAAATGGAACACAGACA
GTGTAGAAGAATTCCTGAGTGAAAAGTTGGAACGCATATAAATCTTGCTTAAATTTTGTCCTATCCTTTTGTTACCTTATC
AAATGAAATATTACAGCACCTAGAAAATAATTTAGTTTTGCTTGCTTCCATTGATCAGTCTTTTACTTGAGGCATTAAATA
TCTAATTAAATCGTGAAATGGCAGTATAGTCCATGATATCTAAGGAGTTGGCAAGCTTAACAAAACCCATTTTTTATAAAT
GTCCATCCTCCTGCATTTGTTGATACCACTAACAAAATGCTTTGTAACAGACTTGCGGTTAATTATGCAAATGATAGTTTG
TGATAATTGGTCCAGTTTTACGAACAACAGATTTCTAAATTAGAGAGGTTAACAAGACAGATGATTACTATGCCTCATGTG
CTGTGTGCTCTTTGAAAGGAATGACAGCAGACTACAAAGCAAATAAGATATACTGAGCCTCAACAGATTGCCTGCTCCTC
AGAGTCTCTCCTATTTTTGTATTACCCAGCTTTCTTTTTAATACAAATGTTATTTATAGTTTACAATGAATGCACTGCATAA
AAACTTTGTAGCTTCATTATTGTAAAACATATTCAAGATCCTACAGTAAGAGTGAAACATTCACAAAGATTTGCGTTAATG
AAGACTACACAGAAAACCTTTCTAGGGATTTGTGTGGATCAGATACATACTTGGCAAATTTTTGAGTTTTACATTCTTACA
GAAAAGTCCATTTAAAAGTGATGTCGACGCG

(b) Sep15ORF-hSECIS

TAGCATGCATCATCATCATCATCATGTGTCTGCTTTTGGGGCAGAGTTTTCATCGGAGGCATGCAGAGAGTTAGGCTTTTC
TAGCAACTTGCTTTGCAGCTCTTGTGATCTTCTCGGACAGTTCAACCTGCTTCAGCTGGATCCTGATTGCAGAGGATGCTG
TCAGGAGGAAGCACAATTTGAAACCAAAAAGCTGTATGCAGGAGCTATTCTTGAAGTTTGTGGATGAAAATTGGGAAGG
TTCCCTCAAGTCCAAGCTTTTGTTAGGAGTGATAAACCCAAACTGTTCAGAGGACTGCAAATCAAGTATGTCCGTGGTTCA
GACCCTGTATTAAAGCTTTTGGACGACAATGGGAACATTGCTGAAGAACTGAGCATTCTCAAATGGAACACAGACAGTGT
AGAAGAATTCCTGAGTGAAAAGTTGGAACGCATATAAATCTTGCTTAAATTTTGTCCTATCCTTTTCCATCGATGGTGCAT
AAAAACTTTGTAGCTTCATTATTGTAAAACATATTCAAGATCCTACAGTAAGAGTGAAACATTCACAAAGATTTGCGTTA
ATGAAGACTACACAGAAAACCTTTCTAAGGATTTGTGTGGATCAGATACATACTTGGCAAATTTTTGAGTTTTACATTCTT
ACAGAAAAGTCCATTTAAAAGTGATGTCGACGCGTATCTCTAGAG

(c) Sep15ORF-chSECIS

TAGCATGCATCATCATCATCATCATGTGTCTGCTTTTGGGGCAGAGTTTTCATCGGAGGCATGCAGAGAGTTAGGCTTTTC
TAGCAACTTGCTTTGCAGCTCTTGTGATCTTCTCGGACAGTTCAACCTGCTTCAGCTGGATCCTGATTGCAGAGGATGCTG
TCAGGAGGAAGCACAATTTGAAACCAAAAAGCTGTATGCAGGAGCTATTCTTGAAGTTTGTGGATGAAAATTGGGAAGG
TTCCCTCAAGTCCAAGCTTTTGTTAGGAGTGATAAACCCAAACTGTTCAGAGGACTGCAAATCAAGTATGTCCGTGGTTCA
GACCCTGTATTAAAGCTTTTGGACGACAATGGGAACATTGCTGAAGAACTGAGCATTCTCAAATGGAACACAGACAGTGT
AGAAGAATTCCTGAGTGAAAAGTTGGAACGCATATAAATCTTGCTTAAATTTTGTCCTATCCTTTTCCATCGATGGACTGA
GCACTGCCGCCCTGTCGCCCGGTCCCGGCAACTGATCACCTGCCAACCCGGCTGGCAACAAGGTTTCCTGTTTGGCGGGTT
GCTGGGTGAGACATGATGGGCGCGGGCTTTAAAGGGCTACAGGCCCTTGGTCCGCCCCGGATGCAAATGATCACCCTAGG
CAACGCCAACTAGGAGGTCATGAGGCTGTGCCGTCGACGCGTATCT
